# Peer Review of “Telerehabilitation for People With Physical Disabilities and Movement Impairment: A Survey of United Kingdom Practitioners”

**DOI:** 10.2196/35852

**Published:** 2022-01-03

**Authors:** Katelyn Brehon

**Affiliations:** 1 Faculty of Rehabilitation Medicine University of Alberta Edmonton, AB Canada

**Keywords:** telerehabilitation, physical disabilities, movement impairment, remote assessments, telehealth, rehabilitation, training, health care practitioners, physiotherapy, occupational therapy

*This is a peer-review report submitted for the paper “Telerehabilitation for People With Physical Disabilities and Movement Impairment: A Survey of United Kingdom Practitioners.”*

## Round 1 Review

### General Comments

This paper [1] adds to the literature base on a very timely and important topic. I appreciated how the qualitative and quantitative results are presented together to highlight each of the major findings. I have provided some comments to help improve the readability and overall quality of the paper, but in general, great work!

### Specific Comments

#### Major Comments

1. In the Discussion regarding survey design and development, there is a discussion about how respondents could only submit responses after every relevant section was filled out. Did each question include an option of prefer not to disclose or open ended response option? If not, consider adding this in the future.

2. When you are including quotes in a manuscript, usually if the quote is less than 40 words, you embed it directly in the text. If it is more than 40 words, you do what you have done currently except with indentation on both sides of the quote.

#### Minor Comments

1. Introduction, first paragraph: “...many people received no face-to-face rehabilitation” should read “many people did not receive any face-to-face rehabilitation”

2. Introduction, second paragraph: “In response, practitioners adapted their practice” should read “In response, practitioners adapted their practices”

3. Introduction, second paragraph: “in the United Kingdom (UK) as worldwide” should read “in the United Kingdom (UK) as well as worldwide”

4. Introduction, third paragraph: “...published guidance, training and support in how to undertake...” should read “...published guidance, training and support on how to undertake...”

5. Methods, second paragraph on design and development: “This process involved informal discussions (e-mail and verbal) with specialists in rehabilitation and physical disabilities, including health and social care practitioners and academics, within and external to the project team” should read “This process involved informal discussions (e-mail and verbal) with specialists in rehabilitation and physical disabilities, including health and social care practitioners and academics within, and external to, the project team”

6. Add info regarding how long the survey took approximately to the Methods

7. In your tables, I suggest aggregating any values that are less than 5, as this could be potentially identifying.

8. Consider reorganizing Figure 2 so that patient benefits and obstacles are side-by-side for ease of comparison

9. You provide examples of the various types of obstacles encountered by practitioners but do not provide examples of organizational and governance obstacles; consider adding some examples of what these included.

10. For Table 4, you list key themes and descriptions, which is great, but this table would benefit from an exemplar quote from each theme.

11. Under “self-perceived confidence and competence,” you report “although most respondents reported that they felt confident in delivering video-based consultations, fewer had confidence in undertaking standardised clinician-rated physical assessments using this method” but do not include any actual numbers from your survey. Please add the numbers in the text rather than leaving it up to the reader to glean numbers from the figure. Additionally, you say that most respondents reported that they felt “confident,” but the questions you are discussing here have to do with proficiency/competence; consider rephrasing.

12. Discussion, paragraph 5: “Understanding the actual versus perceived safety risks, and how risk averseness may impact on the type and quality...” should either read “Understanding the actual versus perceived safety risks, and how risk averseness may impact the type and quality...” or “Understanding the actual versus perceived safety risks, and how risk averseness may have an impact on the type and quality...”
